# Supplementary material for: Mechanisms governing the pioneering and redistribution capabilities of the non-classical pioneer PU.1
Source: Nat Commun. 2020 Jan 21;11:402. doi: 10.1038/s41467-019-13960-2 (PMC6972792; doi:10.1038/s41467-019-13960-2)
Supplement: Supplementary file 7 — Source data [file 41467_2019_13960_MOESM7_ESM.zip › Source_Data/Figure6/Figure6B_MotifScanOutput/ETS1_mutPU.1/homerResults/motif12.similar.html]

motif12

## Information for motif12

G
T
C
A
G
T
A
C
A
G
C
T
T
C
G
A
A
G
T
C
T
C
G
A
G
C
T
A
G
A
C
T
G
A
C
T
G
A
T
C
A
G
T
C
A
G
T
C
  
Reverse Opposite:  

A
C
T
G
A
C
T
G
C
T
A
G
C
T
G
A
C
T
G
A
C
G
A
T
A
G
C
T
A
C
T
G
A
G
C
T
C
T
G
A
A
C
T
G
A
C
G
T
  

|  |  |
| --- | --- |
| p-value: | 1e-149 |
| log p-value: | -3.447e+02 |
| Information Content per bp: | 1.750 |
| Number of Target Sequences with motif | 581.0 |
| Percentage of Target Sequences with motif | 4.31% |
| Number of Background Sequences with motif | 231.8 |
| Percentage of Background Sequences with motif | 0.66% |
| Average Position of motif in Targets | 100.9 +/- 48.0bp |
| Average Position of motif in Background | 92.1 +/- 68.0bp |
| Strand Bias (log2 ratio + to - strand density) | 0.2 |
| Multiplicity (# of sites on avg that occur together) | 1.28 |
| Motif File: | file (matrix) reverse opposite |

### Similar de novo motifs found

|  |  |  |  |  |  |  |  |
| --- | --- | --- | --- | --- | --- | --- | --- |
| Rank | Match Score | Redundant Motif | P-value | log P-value | % of Targets | % of Background | Motif file |
| 1 | 0.848 | C G T A A G T C A G C T C G T A A T G C C T G A G C A T G C A T C G A T T A G C | 1e-145 | -335.348636 | 6.96% | 1.96% | motif file (matrix) |
| 2 | 0.962 | T G C A G T A C G A C T G T C A G A T C T C G A G C T A A G C T G A C T G A T C A G T C G T A C C T G A | 1e-137 | -316.265927 | 4.15% | 0.68% | motif file (matrix) |
| 3 | 0.954 | T G C A G T A C G A C T T G C A G A T C T C G A C G T A A G C T G A C T G A T C A G T C A G T C C T G A A C T G | 1e-134 | -310.664549 | 3.82% | 0.57% | motif file (matrix) |
| 4 | 0.921 | T G C A G T A C G A C T T G C A A T G C T C G A G C T A G A C T C A G T A G T C A G T C | 1e-127 | -293.090315 | 4.39% | 0.87% | motif file (matrix) |
| 5 | 0.758 | C G T A T C A G A C G T A G C T A C T G A C G T C T G A A C T G A C G T | 1e-61 | -141.375720 | 2.50% | 0.60% | motif file (matrix) |
